# Substantial warming of Central European mountain rivers under climate change

**DOI:** 10.1007/s10113-023-02037-y

**Published:** 2023-02-18

**Authors:** Georg H. Niedrist

**Affiliations:** River and Conservation Research, Department of Ecology, University of Innsbruck, Technikerstr. 25, A-6020 Innsbruck, Austria

**Keywords:** Water, Minimum temperature, Maximum temperature, Long-term warming, Heat periods

## Abstract

Water bodies around the world are currently warming with unprecedented rates since observations started, but warming occurs highly variable among ecoregions. So far, mountain rivers were expected to experience attenuated warming due to cold water input from snow or ice. However, air temperatures in mountain areas are increasing faster than the global average, and therefore warming effects are expected for cold riverine ecosystems. In decomposing multi-decadal water temperature data of two Central European mountain rivers with different discharge and water source regime, this work identified so far unreported (a) long-term warming trends (with river-size dependent rates between  +0.24 and  +0.44 °C decade^−1^); but also (b) seasonal shifts with both rivers warming not only during summer, but also in winter months (i.e., up to  +0.52 °C decade^−1^ in November); (c) significantly increasing minimum and maximum temperatures (e.g., temperatures in a larger river no longer reach freezing point since 1996 and maximum temperatures increased at rates between +0.4 and +0.7 °C decade^−1^); and (d) an expanding of warm-water periods during recent decades in these ecosystems. Our results show a substantial warming effect of mountain rivers with significant month-specific warming rates not only during summer but also in winter, suggesting that mountain river phenology continues to change with ongoing atmospheric warming. Furthermore, this work demonstrates that apart from a general warming, also seasonal shifts, changes in extreme temperatures, and expanding warm periods will play a role for ecological components of mountain rivers and should be considered in climate change assessments and mitigation management.

**Supplementary Information:**

The online version contains supplementary material available at 10.1007/s10113-023-02037-y.

## Introduction

Different freshwater habitats around the world are warming with current unprecedented rates since the beginning of observations (O’Reilly et al. 2015; Woolway et al. 2017; Liu et al. 2020) but warming occurs highly variable among ecoregions (Liu et al. 2020). In mountain areas, air temperatures increased faster than the global average (Pepin et al. 2015; Gobiet et al. 2014) and led to accelerated global glacier mass loss (Zemp et al. 2015; Hugonnet et al. 2021) and faster warming of high-altitude streams (Niedrist and Füreder 2021) in the twenty-first century. Water temperature is generally one of the crucial conditions that regulates biological and geochemical structures and processes in freshwaters (Woodward et al. 2010; Bravo et al. 2018; Nagler et al. 2021; Bernabé et al. 2018). In mountain streams, this factor has demonstrated effects on the distribution and range of native freshwater species (Giersch et al. 2015), on the performance and growth of invertebrates (Füreder and Niedrist 2020; Niedrist et al. 2018a), on the formation of thermal niches for non-native species (Khamis et al. 2015; Rahel and Olden 2008), on the survival and fitness of cold-water fish (Young et al. 2018; Al-Chokhachy et al. 2013), or on stream metabolism (Acuña et al. 2008; Ferreira and Canhoto 2015). However, while there is evidence that water temperatures in mountain rivers are changing, only individual studies have so far shown seasonal and annual changes (Michel et al. 2020), or were limited to summer temperatures only (Niedrist and Füreder 2021).

Shorter term temperature dynamics are of importance for aquatic life in rivers (Steel et al. 2012; Ouellet et al. 2020). The extremes of minimum and maximum temperatures attained within any given year can be decisive for a variety of physical and ecological processes in rivers globally (Olden and Naiman 2010; Neuheimer and Taggart 2011) and in mountain regions (Isaak et al. 2012). Minimum and maximum temperatures can regulate the spawning, survival, or the consumption of fish (Martin et al. 2020; Farmer et al. 2015), but also affect the development of fish parasites (Wharton 1999; Kafle et al. 2018). Furthermore, heat events can affect aquatic life and stress especially those that evolved in systems with abundant cold mountain water (McCullough et al. 2009; Réalis-Doyelle et al. 2016). For example, water temperature is negatively correlated to dissolved oxygen, with warmer water physically reducing the solubility and availability of dissolved oxygen for heterotrophic organisms (Jane et al. 2021; Rajesh and Rehana 2022). Most dramatically, heat events (which are expected to occur more frequent and intense (IPCC 2021)) can turn into potential elimination events especially for species requiring high oxygen supply (e.g., salmonid fish (Stehfest et al. 2017)). Despite its importance, the evolution of temperature extremes in mountain rivers has received little attention so far (but see St-Hilaire et al. 2021 for worldwide developments).

Increasing river water temperatures have generally been related to increased air temperatures (Kaushal et al. 2010; Webb et al. 2003), but also climate change–induced hydrological changes (i.e., earlier onset of snowmelt period, decreased summer precipitation, shifting sources due to shrinking glaciers) have been reported to intensify the warming (e.g., Webb and Nobilis 2007; van Vliet et al. 2011; Laghari et al. 2012; Milner et al. 2017). Large mountain rivers are supposed to integrate the mosaic-like thermal situations of all individual tributaries and can thus provide an overall estimate of the thermal status and change for such special ecoregions. Despite the availability of data sets, they have not yet been analyzed at such time scale (decades) and at sub-yearly and daily resolution in the context of climate change.

This study analyzed long-term (>40 years) measurements to evaluate the changes in annual minimum and maximum temperatures of two higher order mountain rivers in the European Alps (using hourly and daily resolved data from automatic gauging stations). Additionally, I related season-specific warming to changes in discharge and measured the prolongation of warm-water periods over the last decades. Since even small thermal changes affect sensitive life phases of aquatic organisms (e.g., reproductive success, egg development, larval growth (Dahlke et al. 2020)), this analysis is not limited to classic heat events. Long-term records of water temperature and discharge from these larger rivers in the European Alps have been decomposed and analyzed under consideration of local air temperature patterns as local climate indicator (similar to Yang and Peterson 2017)). The overall objectives of this work were first, to quantify the overall warming of mountain rivers, second to identify month-specific changes in water temperature (adapted from lake patterns Niedrist et al. 2018b; Winslow et al. 2017), and third to quantify changes in the exceeding of temperature levels over the period of 43 years. It was assumed that independent of changes in monthly patterns and random fluctuations, the overall temperature of Alpine rivers underwent a statistically significant increase during the last decades similar to other rivers in Central Europe (e.g., Michel et al. 2020; Niedrist and Füreder 2021). Furthermore, I hypothesized that water temperature extremes increased over time as response to increased maximum air temperatures in the same ecoregion (Gobiet et al. 2014).

## Methods

### Study rivers

**Fig. 1:**
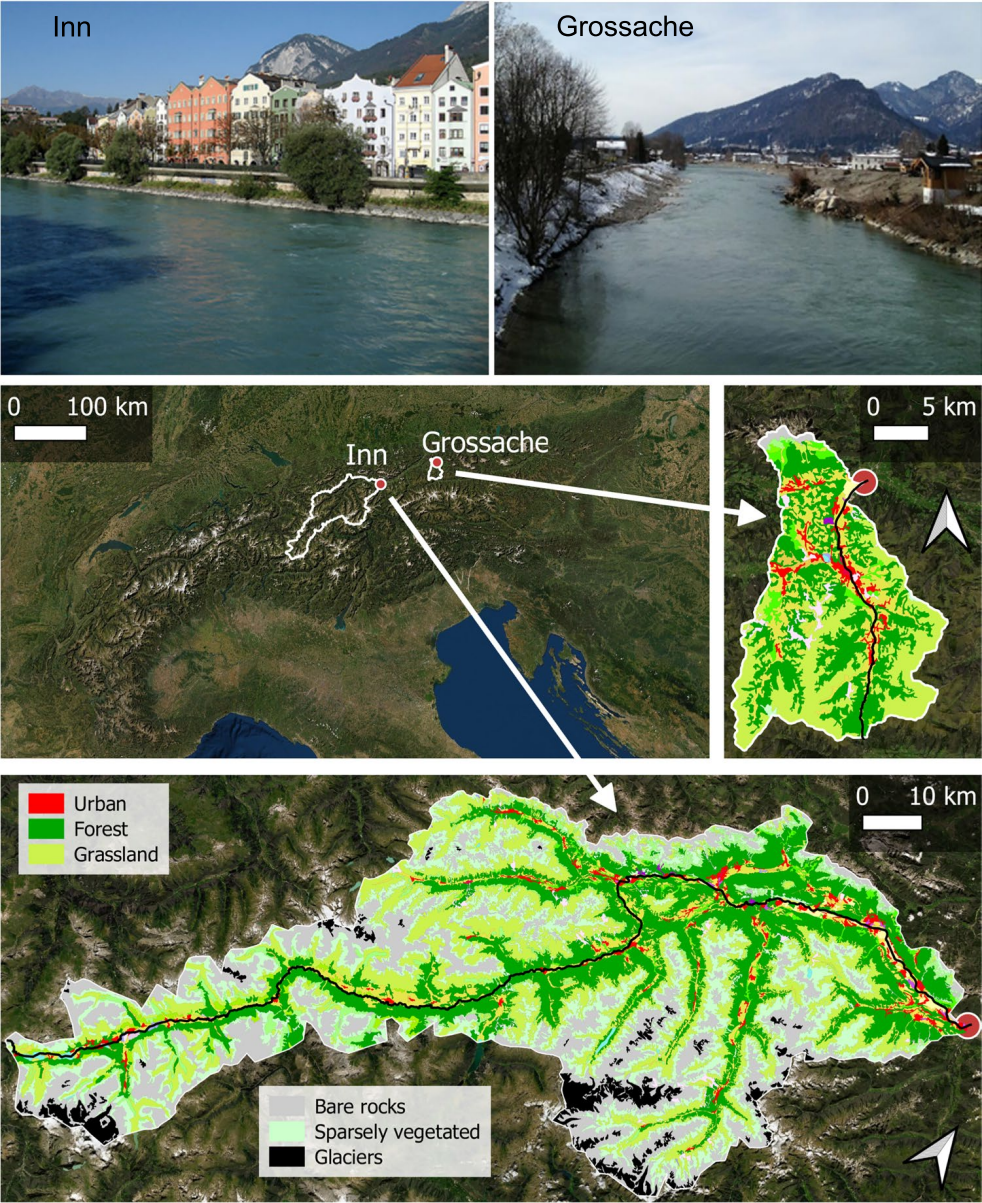
Study sites and catchments in the European Alps with indicated CORINE land-cover types (from 2018; red, urban areas; greens, forest and grassland; blue/gray, sparsely vegetated areas/rocks; black, glaciers and perpetual snow). The red dots represent the position of the monitoring station

River *Inn* (river A) and river *Grossache* (river B) are inner-alpine rivers in the European Alps with (A) and without (B) input of glacial meltwater (Fig. 1). Until the monitoring stations, the river *Inn* flows through parts of Switzerland and Austria and drains a catchment of 5771 km^2^, of which most area is located in the alpine climate zone (Bobek et al. 1971) with contributions of glaciated and non-glaciated alpine and mountainous sub-catchments. In contrast, the river *Grossache* drains a smaller catchment (701 km^2^) at lower altitudes (mountain climate zone) without glaciers or larger rock areas in its catchment.

### Data sources

River discharge and water temperature data were obtained from the automatic gauging stations (Fig. 1) in Innsbruck (for river *Inn*, located at 565 m a.s.l.) and in Koessen (for river *Grossache*, located at 589 m a.s.l.), which monitor discharge and water temperature at daily intervals since 1977 (for *Inn*) and at hourly intervals since 1997 (for both study rivers). Local air temperature data were obtained as monthly mean air temperatures from meteorological stations close to the hydrological sites and obtained by the HISTALP project (https://www.zamg.ac.at/histalp/index.php). Land-cover data were obtained from freely available CORINE Land Cover (CLC) data. Digital elevation data (retrieved from the EU-funded COPERNICUS platform) is based on a 25-m resolution.

### Analysis and statistics

River water temperature data were summarized to monthly minimum, mean, and maximum temperatures, and yearly minimum and maximum temperatures were extracted for each year. Discharge, air temperature, and summarized water temperature data were then resolved into long-term trends (using a 12-month moving average), the annual cycle (averaged monthly patterns), and the random (non-cyclic) variation, using a classical seasonal decomposition of the time-series from the *stats* package (R). The extracted long-term trends, month-specific trends, and changing yearly minimum and maximum temperatures over time were described using linear regressions. To assess and describe the lengthening of periods of different warm periods, the days on which the water temperatures exceeded certain values (10–18 °C) were summed up and linked to the study years using linear models.

Used models were checked for normality (Kolmogorov–Smirnov), autocorrelation (pacf-function), influential measurements (leverage and studentized residuals), and heteroscedasticity of residuals (Levene-test). The presented plots and analyses were all compiled in R v 4.1.2. (R Core Team 2021) using the packages zoo (Zeileis and Grothendieck 2005) and ggplot2 (Wickham 2016).

Hydrological catchments were delineated using QGIS (QGIS Development Team 2009) with the GRASS GIS add-on (GRASS Development Team 2017).

## Results

### Long-term changes in ambient air temperature

At both meteorological stations, mean air temperature significantly increased within the last century (1910–2021) with mean rates of  +0.41 °C decade^−1^ close to river *Inn* and  +0.47 °C decade^−1^ close to river *Grossache* (*F*>48, *p* < 0.001, Fig. S1) and is highly correlating with nearby water temperatures at river Inn (*R*^2^ = 0.96, Fig. S1C) and river Grossache (*R*^2^ = 0.95, Fig. S1D).

### Long-term and month-specific warming of river temperatures

**Fig. 2:**
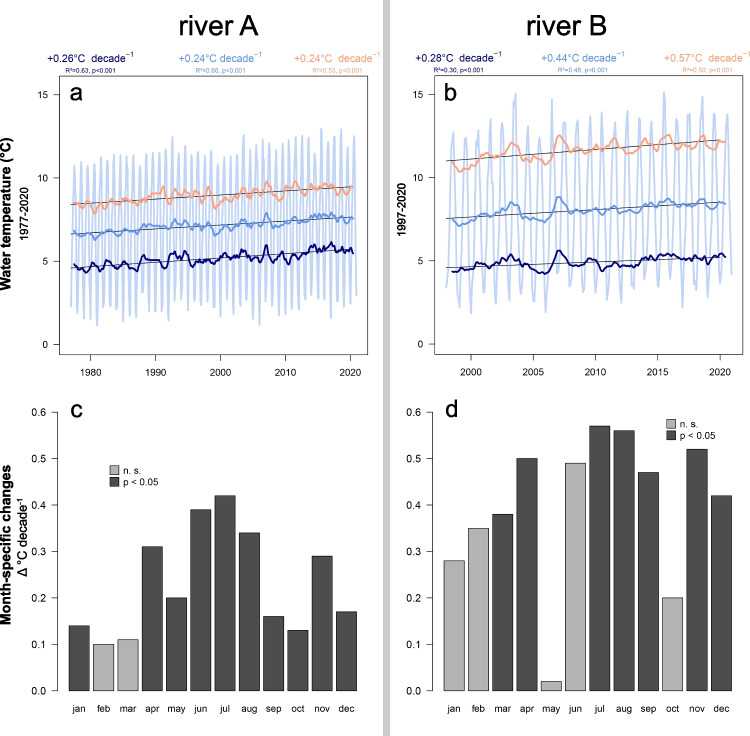
Long-term changes (**a** and **b**) and month-specific warming (**c** and **d**) of water temperatures in river A — *Inn* (left side, from 1977 to 2020) and river B — *Grossache* (right side, from 1998 to 2020). **a** and **b**: Moving average (12-month window) of monthly mean (blue), minimum (dark blue), and maximum (orange) temperatures with the observed monthly mean temperatures in the background (light blue). **c** and **d**: Significant (dark-gray) and non-significant (light-gray) linear regression slopes of month-specific mean water temperatures visualize and quantify the month-specific warming

In river *Inn*, monthly mean water temperature increased significantly at an average rate of +0.24 °C decade^−1^ (*R*^2^ = 0.64, *p* < 0.001; Fig. 2a), corresponding to an increase from 6.6 °C (average during 1977–1981) to 7.6 °C (average during 2016–2020) over the study period (see Fig. S2 for monthly variation of the river temperatures). Similarly, increasing rates of monthly minimum (+0.26 °C decade^−1^) and maximum temperatures (+0.24 °C decade^−1^) differed significantly from zero (*R*^2^ > 0.5, *p* < 0.001; Fig. 2a).

In river *Grossache*, monthly mean temperature increased at a higher rate (+0.44 °C decade^−1^, *R*^2^ = 0.48, *p* < 0.001), together with the monthly minimum and maximum temperatures (+0.28 °C and  +0.57 °C decade^−1^ for minimum and maximum temperatures, respectively; Fig. 2b).

The decomposition of temperature data indicated differential month-specific increases of river temperatures in both rivers (Fig. 2c, d). Largest and significant increases (*p* < 0.001) in both rivers occurred in the summer months June (+0.40 °C and  +0.49 °C decade^−1^ for river *Inn* and *Grossache*, respectively), July (+0.42 °C and  +0.57 °C decade^−1^), and August (+0.34 °C and  +0.56 °C decade^−1^) and in the winter months November (+0.29 °C and  +0.52 °C decade^−1^) and December (+0.17 °C and  +0.42 °C decade^−1^). Additionally, river *Grossache* showed significant increases in March (+0.38 °C decade^−1^), April (+0.50 °C decade^−1^), and September (+0.47 °C decade^−1^) temperatures, while river *Inn* warmed significantly also in the months January (+0.14 °C decade^−1^) and April (+0.31 °C decade^−1^). Month-specific warming rates for the other months were all positive, but not significantly different from zero (*p* > 0.05, Fig. 2c, d).

### Long-term and month-specific changes in river discharge

Generally, river *Inn* drains a larger catchment and reaches a much higher maximum discharge during summer (408 m^3^ s^−1^, averaged for 2012–2016) than river *Grossache* (45.3 m^3^ s^−1^, averaged for 2012–2016). The overall discharge of the river *Inn* remained constant over time (+1 m^3^ decade^−1^, *R*^2^ = 0.01, *p* = 0.012), but the phenology changed from 1977 to 2020 with runoff increasing from October to May and decreasing in summer (June–September, Fig. S3b). Discharge during summer decreased with rates up to  −14.2 m^3^ decade^−1^ in July (*p* = 0.002, Fig. S3B), but increased during the rest of the year (October to May, with a maximum rate of  +6.6 m^3^ decade^−1^ for November discharge, Fig. S3B).

### Changes in yearly maximum and minimum water temperatures

**Fig. 3:**
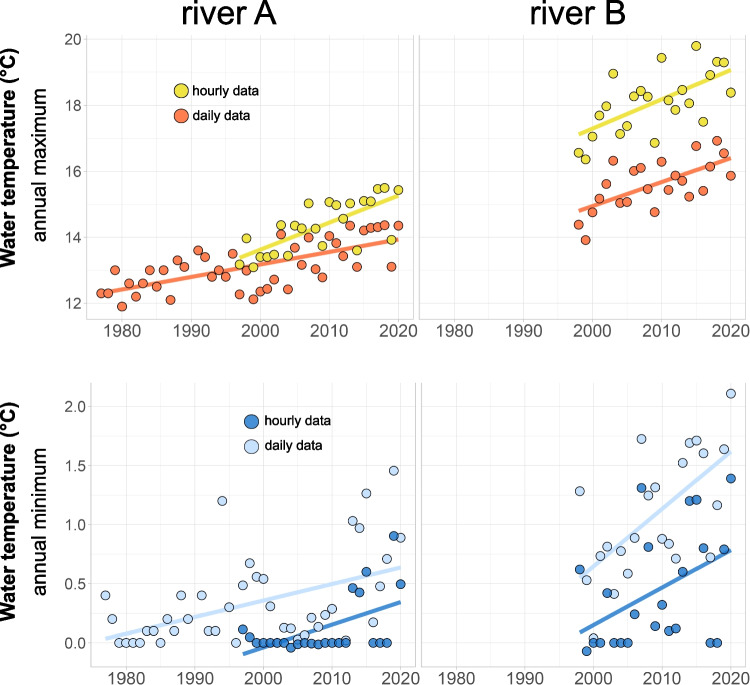
Annual maximum (**A** and **B**) and minimum (**C** and **D**) daily mean and hourly water temperatures of river A - *Inn* from 1977 to 2020 (left) and river B - *Grossache* from 1997 to 2020 (right). Linear models (lines) fit the multi-annual increases

Continuous water temperature records showed a substantial increase in annual maximum and minimum temperatures in both rivers over the last decades (Fig. 3). In this period, both rivers’ water temperature extremes (maximum and minimum) increased steadily, not-withstanding inter-annual variability. On average, the yearly maximum of daily mean temperatures increased by 0.4 °C decade^−1^ for river A - *Inn* (linear model, *R*^2^ = 0.46, *p* < 0.001, Fig. 3A) and by 0.7 °C decade^−1^ for river B - *Grossache* (linear model, *R*^2^ = 0.43, *p* < 0.001, Fig. 3B). Noteworthy is the sharp increase of minimum and maximum daily water temperatures during the last decade (2010–2020) (Fig. 3) that correlated with increases in local air temperatures (*r* = 0.48 and 0.71 for river A and river B, respectively). The five highest daily water temperatures in the *Inn* river (>14.3 °C) were all measured in the last 7 years (2013–2020; Fig. 3A). While the temperatures in the beginning of the study period (1977–1986, 10 years) reached a maximum of 13 °C, the highest values in the last 10 years (2011–2020) were all above 13 °C and in most years higher than 14.3 °C.

Annual minimum temperatures remained low for decades (multiannual average remained below 0.5 °C, Fig. 3C, D) in both study rivers but increased with rates of 0.1 °C (river A - *Inn*, *p* < 0.05) and 0.5 °C decade^−1^ (river B - *Grossache*, *p* < 0.05). While annual minimum temperatures remained low for decades, the coldest temperatures were much higher within the last decade in both rivers (Fig. 3C, D). In addition to daily averaged temperature patterns, hourly data reached higher maximum temperatures (15.5 °C and 19.8 °C in river *Inn* and *Grossache*, respectively) and increased over time in both rivers with similar rates (+0.8 °C and  +0.9 °C decade^−1^, Fig. 3A, B).

### Exceeding critical temperature levels in Alpine streams

The number of days, on which hourly mean water temperatures exceeded certain limit values (10 °C, 11 °C, 12 °C, 13 °C, 14 °C, 15 °C, 16 °C, 17 °C, and 18 °C), increased significantly in both study rivers, although higher values were reached in river B. The absolute number of days was negatively related to the level of the threshold (i.e., while 10 °C daily mean temperature was exceeded on 88–164 days in river A or 145–187 in river B, higher temperatures (e.g., 13 °C) have been exceeded on less days (on 1–52 days in river A or 66–123 days in river B, Fig. 3)).

**Fig. 4:**
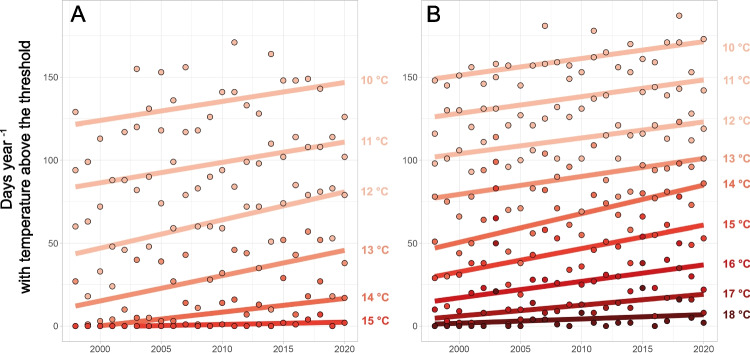
Number of days per year on which the hourly mean water temperature of rivers **A** and **B** exceeded the limits 10 °C, 11 °C, 12 °C, 13 °C, 14 °C, 15 °C, 16 °C, 17 °C, or 18 °C. The lines (fitting linear models) indicate the corresponding trend over the period from 1998 to 2020

The increases in days over the study period (1998 to 2020) were highest for exceeding 12 °C in river *Inn* and 14 °C in river *Grossache* with the same rate of +17 days decade^−1^ (*β* = 16.9, *R*^2^ = 0.36, *p* = 0.002 and *β* = 17.2, *R*^2^ = 0.40, *p* = 0.001, for both rivers respectively). Hourly mean water temperatures did not exceed 16 °C in river *Inn* (Fig. 4A) or 20 °C in river *Grossache* (Fig. 4B). The highest temperatures (15.5 °C and 19.8 °C in river *Inn* and *Grossache*, respectively) were reached during end of July and beginning of August.

## Discussion

The climate crisis is impacting ecosystems globally (Malhi et al. 2020) and is also affecting mountain areas and mountain ecosystems (Hock et al. 2019). This work investigated how water temperature of selected Alpine rivers changed during the last decades (since the 1970s), a period when air temperature rose considerably, glaciers receded at unprecedented rates (Gobiet et al. 2014; Sommer et al. 2020; Hugonnet et al. 2021), and precipitation in the form of snow decreased (Matiu et al. 2021). Warming of such cold-water ecosystems had been quantified previously (e.g.,Michel et al. 2020; Niedrist and Füreder 2021), but this study is the first to investigate evolutions of minimum and maximum temperatures and month-specific warming of Alpine rivers using long-term and fine-scaled observational data from typical higher-order mountain rivers. The main findings are the quantification of average warming rates between +0.24 and +0.45 °C decade^−1^ and the identification of month-specific responses to climatic warming during the last decades with significant warming rates not only during summer but also in winter months.

The warming rates identified in both streams were different (+0.24 vs. +0.45 °C decade^−1^) and most probably related to the size of the rivers and/or the water source contribution (river Inn has glacial meltwater input, 3.5% of the catchment [=185 km^2^] are glaciers). Glacier meltwater input is generally lowering river temperature (Williamson et al. 2019), but this study showed that the mixing with non-glacial input still results in a net warming of 0.24 °C decade^−1^. It is therefore foreseeable that the rate of river warming will increase somewhat as glaciers keep retreating globally (Hugonnet et al. 2021) and regionally in the European Alps (Sommer et al. 2020). The delimited slopes of water temperature (°C decade^−1^) of the studied rivers are similar to those reported for comparable river types (Swiss mountain rivers (Michel et al. 2020)) but lower than in low-order high altitude streams (Niedrist and Füreder 2021).

Despite the absolute thermal differences between the studied rivers and the overall warming rates, the monthly patterns of warming were similar and also comparable to the observed month-specific warming in mountain lakes (Niedrist et al. 2018b) and revealed not only warming during summer, but also during autumn/winter. The warming during these colder months is likely to be as important as the impacts of summer-warming, since many biological processes (e.g., insect emergence, salmonid spawning and hatching) depend on water temperature patterns in this season (Durance and Ormerod 2007; Rooke et al. 2020; Tao et al. 2018; Réalis-Doyelle et al. 2016). Hence, increasing water temperatures during autumn/winter months need to be considered in fishery management of mountain rivers.

This study identified that yearly minimum temperatures in usually cold Alpine rivers have been found to no longer reach 0 °C. The daily mean temperatures shifted to beyond 1 or 1.5 °C within the last decade and are expected to further increase at high rates. More than changing the phenology of benthic invertebrate and fish species (Schütz and Füreder 2019; Crozier et al. 2008), further increasing river minimum temperatures will result in freeze-free years and potentially consequential changes in the phenology, geographical distribution, or the density of parasites and pathogenic viruses/bacteria (Tops et al. 2006).

Yearly maximum temperatures regularly exceed 15 °C (in the large mountain river) and 19 °C (in the river without glacial contribution) and are expected to further increase at high rates. Although below the lethal maximum water temperature of salmonid fish (Pfeiler and Kirschner 1972; Zaugg and Wagner 1973), such high temperatures (>16 °C) have been reported to induce cellular and endocrine stress responses and thus limit growth (Chadwick and Mccormick 2017). Furthermore, periods with higher temperatures are expected to favor the emergence and density of (fish) parasites (Macnab and Barber 2012).

Distinct species of mountain aquatic communities differ in their temperature preferences to warming (e.g., invertebrates (Niedrist and Füreder 2016)); thus, the response to increasing temperatures in the same river might differ between species and those adapted to cold temperatures during early life stages might be most sensitive to warming waters (e.g., salmonid fish, (Young et al. 2018)). Furthermore, a general warming can alter phenotypic cycles and sex determinations of biological communities (Valenzuela et al. 2019; Mitchell and Janzen 2010), of which examples had also been reported in Alpine regions (i.e., an observed shifted spawning of European graylings led to a male-biased population sex ratio in Switzerland (Wedekind and Küng 2010; Wedekind et al. 2013)), will reduce the expansion range of cold-water species (Jacobsen and Dangles 2017), allow immigration of non-native species (Niedrist et al. *under review*), or enhance the expansion and densities of parasites or parasite hosts (Macnab and Barber 2012; Tops et al. 2006). Overall, the observed thermal shifts and the estimated changes (IPCC 2021) are expected to cause shifts within aquatic communities with expansions for temperate species and range contractions for cold-water species (Hurford et al. 2019; Van Zuiden et al. n.d.), finally advancing homogenizations of aquatic communities. As higher temperatures are also positively linked to in-stream production (Downing 2014) and leaf litter breakdown rates (Follstad Shah et al. 2017), they are expected to affect the provision and recycling of nutrients and mountain river ecosystem functioning.

The continuation of atmospheric warming and the expected acceleration in mountain regions in particular will persistently change aquatic ecosystems and economic habits such as fishery (e.g., warming will increase stress for important regional salmonid resources and hamper ongoing efforts to recover native fish species). Considering the here reported warming of mountain rivers, strategic conservation efforts and climate adaptation measures are needed in these ecosystems.

## Conclusion

This study identified climate warming–induced changes in month-specific mean and minimum/maximum temperatures in higher order mountain streams. The identified extension of warm periods and the significant warming of mountain river temperatures, and more precisely the detected winter-warming effect, is expected to affect biological processes and cold-water organisms (e.g., spawning success, hatching time, parasites). This work suggests that this autumn to early winter warming and the increases of yearly minimum temperatures lead to shifts in river phenology, which might affect biological life cycles (e.g., most salmonid fishes lay eggs/spawn in autumn to early winter). Increasing maximum temperatures will additionally directly stress cold-water organisms (Réalis-Doyelle et al. 2016; McCullough et al. 2009). Given the importance of water temperature for the ecology or the metabolism of rivers (Woodward et al. 2010; Attermeyer et al. 2021), and the fact that temperatures are increasing rapidly worldwide (IPCC 2021), our findings of winter warming and increasing maximum/minimum temperatures are important to understand the thermal dynamics of mountain rivers and the consequences of climatic changes on river ecosystems in general.

## Supplementary Information

Below is the link to the electronic supplementary material.

Supplementary file1 (PDF 944 KB)

## Data Availability

The raw data used in this study is obtained from a governmental source, the elaborated data is available upon request for research purposes by contacting the corresponding author.

## Declarations

This article does not contain any studies with human or animal participants performed by the author.
