# Supplementary material for: Substantial warming of Central European mountain rivers under climate change
Source: Reg Environ Change. 2023 Feb 18;23(1):43. doi: 10.1007/s10113-023-02037-y (PMC9938829; doi:10.1007/s10113-023-02037-y)
Supplement: Supplementary file 1 — Supplementary file1 (PDF 944 KB) [file 10113_2023_2037_MOESM1_ESM.pdf]

Regional Environmental Change

Supplementary results

of the research article

Substantial warming of Central European mountain rivers under climate change

Niedrist G. H.

University of Innsbruck, Department of Ecology, River and Conservation Research, Innsbruck, Austria;

ORCID; correspondence: Georg.Niedrist@uibk.ac.at

Content

Fig. S1 – Increasing trend of local air temperatures and highly correlation between air and water temperatures.

Fig. S2 – Seasonal variation of monthly mean water temperature along the study period. Numbers indicate the months of the year (e.g., January to December = 1 – 12).

Fig. S3 – Long-term and seasonal changes of discharge in a mountain river (Inn, Austria).

Supplementary statistical results

Figure S1

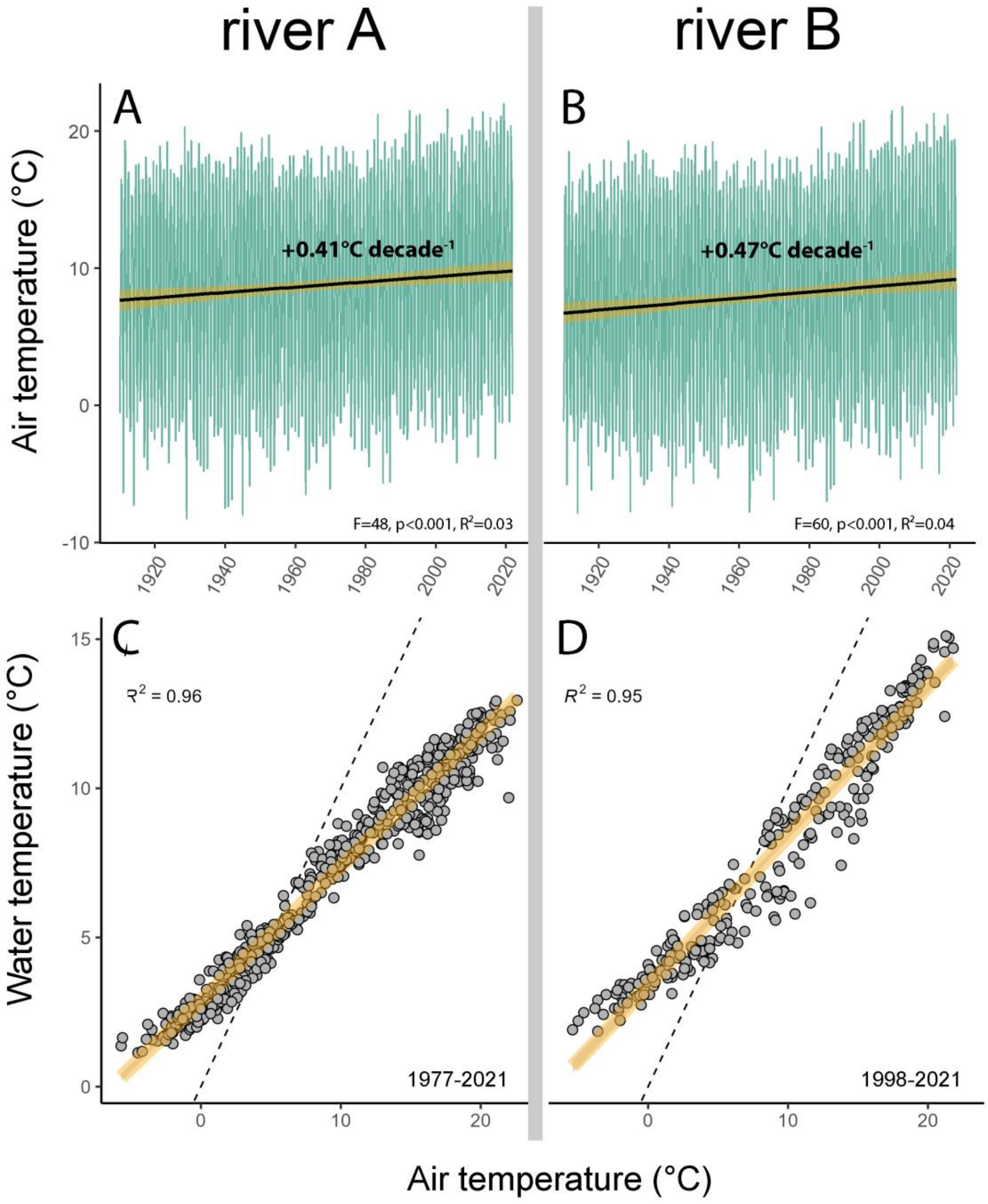

Fig. S1. Twelve-month moving average of monthly mean air temperatures close to study rivers (A and B, green curves) with overall linear models from 1910-2020 (black line) and standard error (orange area). Local air temperature The local air temperatures correlate with the temperatures of the study rivers (C and D, yellow lines indicate the temperature links, dashed lines are the 1:1 relationship).

Figure S2

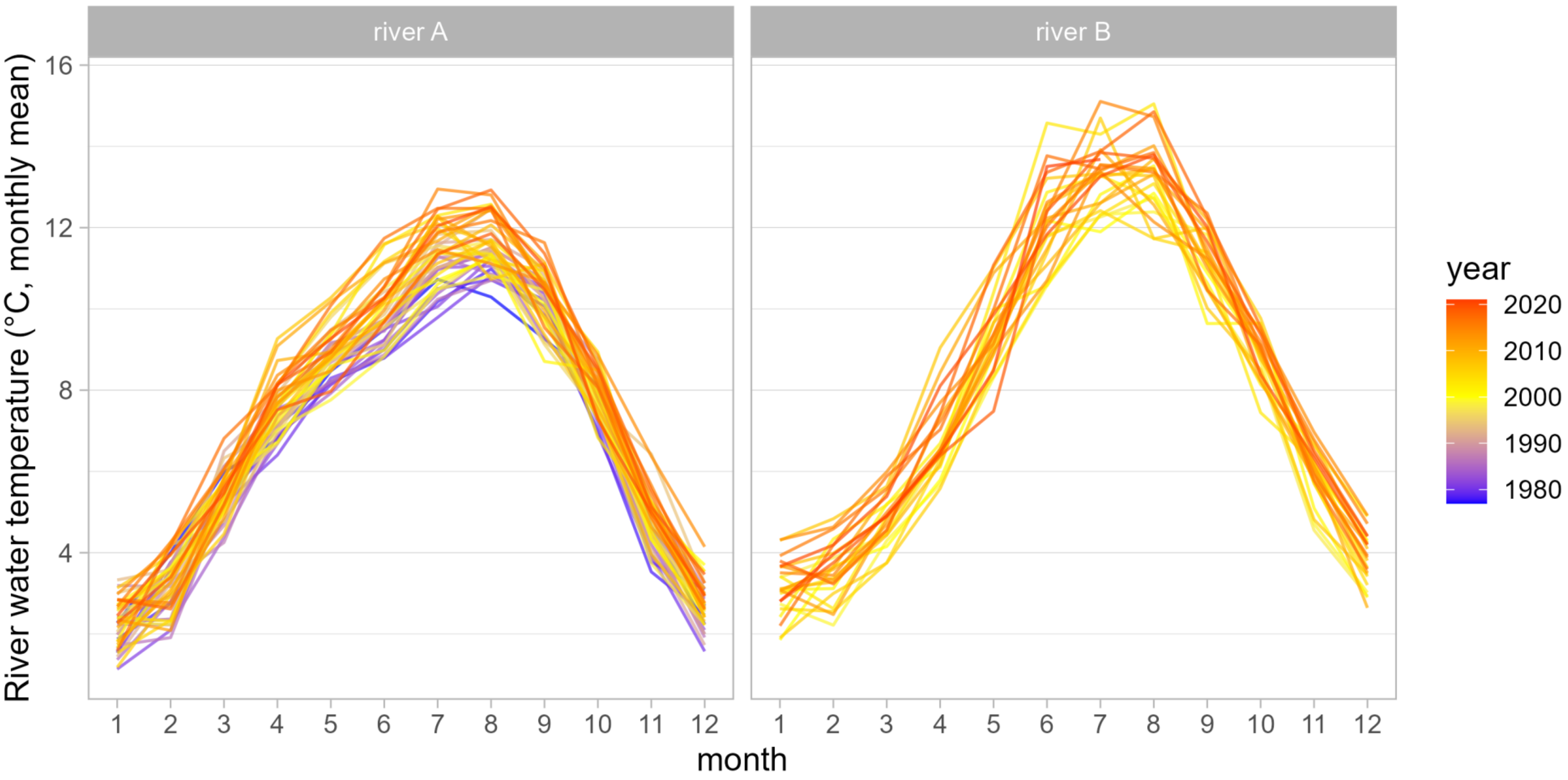
river A
river B
River water temperature (°C, monthly mean)
16
12
8
4
1 2 3 4 5 6 7 8 9 10 11 12
1 2 3 4 5 6 7 8 9 10 11 12
month
year
2020
2010
2000
1990
1980

Figure S3

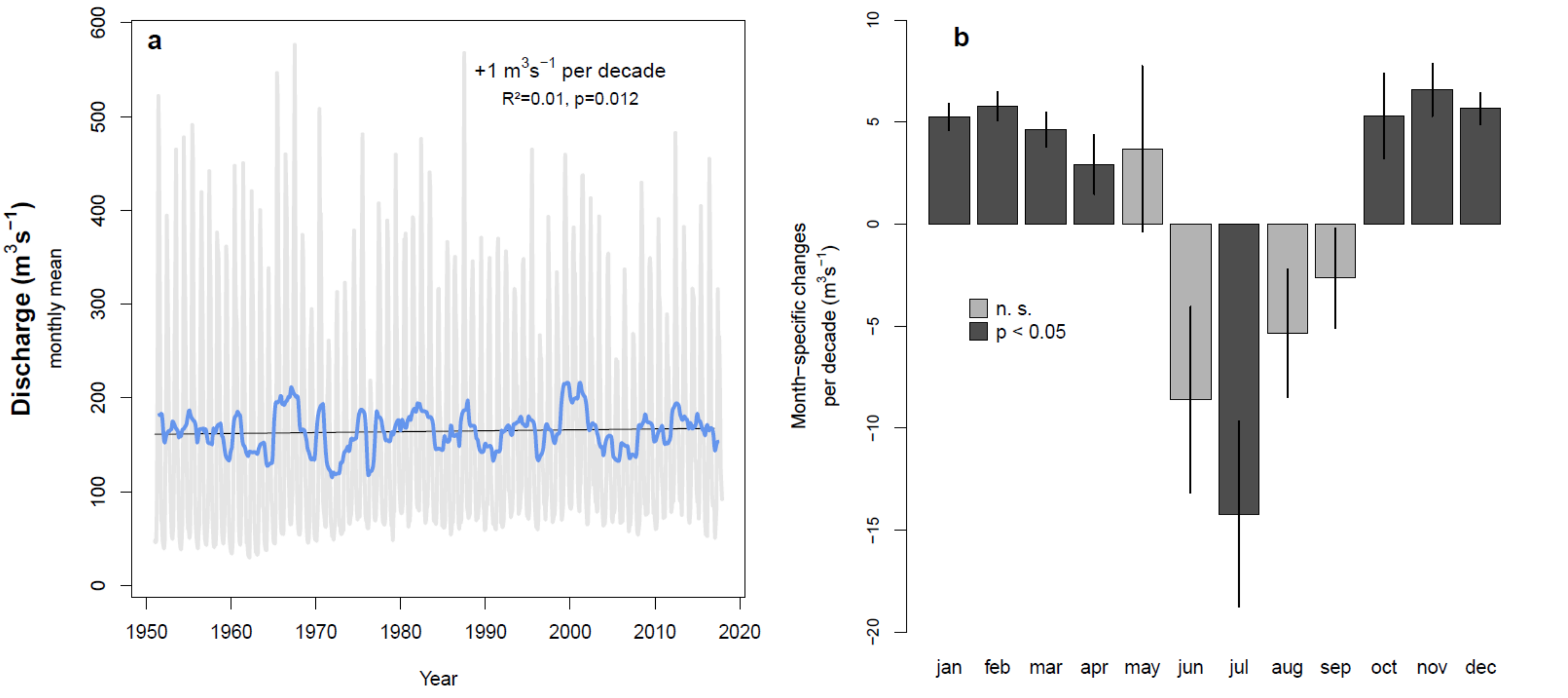

Fig. S3. Long-term changes (left) and month-specific warming (right) of discharge in river Inn from 1977 to 2020): Moving average (52-month) of monthly mean (light blue) with the observed monthly discharge in the background (grey); Significant (dark-grey) and non-significant (light-grey) bars represent linear regression slopes of month-specific mean discharge over the study period.

Supplementary statistical results

The assumptions for using linear models to describe changes in extracted long-term trends following the time-series-decompositions were met for discharge, air temperature and water temperature datasets. The residuals were normally distributed (Kolmororov-Smirnov, $p>0.05$), the residuals had a constant variance ('homoscedastic residuals', Levene-test, $p>0.05$), with negligible lag3 autocorrelation (*pacf*, partial autocorrelation factor <0.2).
